# Platelet proteome reveals novel pathways of platelet activation and platelet-mediated immunoregulation in dengue

**DOI:** 10.1371/journal.ppat.1006385

**Published:** 2017-05-19

**Authors:** Monique Ramos de Oliveira Trugilho, Eugenio Damaceno Hottz, Giselle Villa Flor Brunoro, André Teixeira-Ferreira, Paulo Costa Carvalho, Gustavo Adolfo Salazar, Guy A. Zimmerman, Fernando A. Bozza, Patrícia T. Bozza, Jonas Perales

**Affiliations:** 1 Laboratório de Toxinologia, Instituto Oswaldo Cruz, Fundação Oswaldo Cruz, Rio de Janeiro, Brazil; 2 Centro de Desenvolvimento Tecnológico em Saúde (CDTS), Fundação Oswaldo Cruz, Rio de Janeiro, Brazil; 3 Laboratório de Imunofarmacologia, Instituto Oswaldo Cruz, Fundação Oswaldo Cruz, Rio de Janeiro, Brazil; 4 Laboratório de Análise de Glicoconjugados, Departamento de Bioquímica, Instituto de Ciências Biológicas, Universidade Federal de Juiz de Fora (UFJF), Juiz de Fora, Minas Gerais, Brazil; 5 Laboratório de Proteômica e Engenharia de Proteínas, Instituto Carlos Chagas (ICC), Fiocruz, Curitiba, Paraná, Brazil; 6 Computational Biology Group, Faculty of Health Sciences, University of Cape Town, Cape Town, South Africa; 7 Department of Internal Medicine, University of Utah, Salt Lake City, UT, United States of America; 8 Instituto Nacional de Infectologia Evandro Chagas (INI), Fundação Oswaldo Cruz, Rio de Janeiro, Brazil; Purdue University, UNITED STATES

## Abstract

Dengue is the most prevalent human arbovirus disease worldwide. Dengue virus (DENV) infection causes syndromes varying from self-limiting febrile illness to severe dengue. Although dengue pathophysiology is not completely understood, it is widely accepted that increased inflammation plays important roles in dengue pathogenesis. Platelets are blood cells classically known as effectors of hemostasis which have been increasingly recognized to have major immune and inflammatory activities. Nevertheless, the phenotype and effector functions of platelets in dengue pathogenesis are not completely understood. Here we used quantitative proteomics to investigate the protein content of platelets in clinical samples from patients with dengue compared to platelets from healthy donors. Our assays revealed a set of 252 differentially abundant proteins. *In silico* analyses associated these proteins with key molecular events including platelet activation and inflammatory responses, and with events not previously attributed to platelets during dengue infection including antigen processing and presentation, proteasome activity, and expression of histones. From these results, we conducted functional assays using samples from a larger cohort of patients and demonstrated evidence for platelet activation indicated by P-selectin (CD62P) translocation and secretion of granule-stored chemokines by platelets. In addition, we found evidence that DENV infection triggers HLA class I synthesis and surface expression by a mechanism depending on functional proteasome activity. Furthermore, we demonstrate that cell-free histone H2A released during dengue infection binds to platelets, increasing platelet activation. These findings are consistent with functional importance of HLA class I, proteasome subunits, and histones that we found exclusively in proteome analysis of platelets in samples from dengue patients. Our study provides the first in-depth characterization of the platelet proteome in dengue, and sheds light on new mechanisms of platelet activation and platelet-mediated immune and inflammatory responses.

## Author summary

Dengue is the most frequent hemorrhagic viral disease and re-emergent infection in the world. Recent decades were marked by a progressive global expansion of the infection including a higher frequency of severe dengue. Currently there is no effective vaccinal coverage or specific therapies, while efforts aimed at vector control have failed to stop the progression of epidemics and expansion of the disease. An increased understanding of the molecular physiology is of paramount importance for the establishment of new therapeutic targets and better clinical management. Dengue fever is characterized by thrombocytopenia and vascular leak. Although thrombocytopenia is a hallmark of dengue, the molecular phenotype and activities of platelets in the pathogenesis of dengue is not well elucidated. This work characterizes the proteome of platelets isolated from patients with dengue and includes validation of functionally-linked protein networks that we identified, using samples from a larger cohort of dengue patients. Moreover, *in vitro* experiments revealed activities of platelets that have recognized importance to dengue pathogenesis, including chemokine release, antigen presentation, and proteasome activity. Finally, our results identify circulating histones as a novel mechanism of platelet activation in dengue. These findings provide new evidence for platelet immune activities in dengue illness, and mark an advance in the understanding of this disease.

## Introduction

Dengue is an infectious disease caused by four antigenically-related serotypes of dengue virus (DENV-1 to -4). It is the most frequent hemorrhagic viral disease and re-emergent infection in the world, with over 2.5 billion people living in high-risk transmission areas and more than 90 million apparent infections registered annually [1,2,3]. DENV-infection may present distinct clinical manifestations varying from mild self-limited dengue fever to life-threatening severe dengue, a syndrome associated with increased vascular permeability, hypovolemia, hypotension and eventually organ dysfunctions and shock [1,3,4]. Thrombocytopenia is a common feature in dengue syndromes and the drop in platelet counts is temporally associated with hemodynamic instability and progression to severity [5,6,7,8]. Nevertheless, the roles played by platelets in dengue pathogenesis remain poorly understood.

Platelets are highly specialized effector cells in hemostasis. Besides well-known hemostatic activities, newly-recognized platelet functions mediate both immune protective activities, including pathogen sensing and host responses, and inflammatory and immune injury to the host [9,10,11]. It is increasingly recognized that activated platelets have a repertoire of mechanisms for immune effector activity including release of cytokines and interaction with leukocytes [9,12,13]. As an example, it was recently shown that platelets are able to process and present antigens derived from exogenous plasmodial proteins in a fashion involving major histocompatibility complex (MHC) (also termed human leukocyte antigen–HLA) class I [14]. New discoveries of platelet biology of this type suggest that knowledge of global changes in platelet proteome, phenotype and function in dengue infection may contribute to a broader understanding of the pathobiology of dengue disease, as in other infections (9–11).

DENV has been detected in circulating platelets from infected patients [15,16]. *In vitro* studies demonstrated DENV binding mechanisms and viral protein synthesis by platelets [17,18]. We have recently shown that platelet activation contributes to altered vascular barrier integrity and innate immune activation in dengue [19,20]. Nevertheless, the mechanisms underlying platelet activation and function in dengue patients remain incompletely understood. Here we describe a shotgun proteomic approach intended to identify and quantify changes in platelet protein abundance in patients with dengue in comparison to that in platelets from healthy volunteers using a label-free mass spectrometry (MS)-based quantification. We found 252 differentially abundant proteins among dengue and control platelets. After an *in silico* biological process characterization, we observed high significance in proteins belonging to antigen processing and presentation, platelet activation, and immune and inflammatory responses activities. In parallel studies, platelet activation and secretion of stored chemokines was verified in an expanded cohort of dengue patients. DENV infection of platelets from healthy volunteers *in vitro* also induced platelet activation and chemokines secretion. In addition, DENV infection enhanced platelet expression of HLA class I and its surface display through mechanisms depending on proteasome activity. Interestingly, our proteome approach detected histones, a group of proteins with diverse biologic activities, exclusively in platelets from dengue-infected patients. Our findings indicate that platelets sequester circulating histones released during dengue infection, contributing to platelet activation. Taken together, our results indicate that the platelet proteome is altered in a functionally-significant fashion in dengue, identify new pathways involved in platelet activation in dengue infection, and provide new insights into dengue pathogenesis.

## Results

### Proteomic analysis of platelets from dengue patients and healthy volunteers

In order to investigate in-depth global changes in the platelet proteome during dengue infection, platelets (isolated with depletion of CD45+ leukocytes) from eight patients with clinical characteristics of having dengue were lysed in RapiGest SF (Waters) and prepared for proteomic analysis as described in the methods. After serological and molecular diagnostic confirmation through detection of IgM antibodies against dengue E protein and viral genome in patient plasma, two dengue-negative patients were excluded and samples from six dengue-confirmed patients (whose characteristics are presented in **Table 1**) were applied to a shotgun proteomic approach as follows.

**Table 1:** Characteristics of dengue-infected patients and healthy volunteers in proteome and validation cohorts.

	Proteome		Validation*		P**
	Control (5)	Dengue (6)	Control (22)	Dengue (36)	
Age, years	31 (29–34)	37 (28–44)	31 (29–34)	33 (28–42)	0.759
Gender, male	2 (40%)	3 (50%)	11 (50%)	20 (55.5%)	0.400
Platelet count, x1,000 /mm^3^	–	120 (94–171)	–	109 (80–161)	0.827
Hematocrit, %	–	44.5 (41–45.3)	–	41.8 (39.7–45)	0.569
Albumin, g/dL	–	3.7 (3.5–4.1)	–	3.6 (3.3–3.7)	0.291
TGO/AST, IU/L	–	83.4 (36–177)	–	53.5 (32–121)	0.508
TGP/ALT, IU/L	–	107.5 (55–249)	–	68 (46.2–124)	0.326
Mild dengue		2 (33.3%)		17 (47.2%)	0.673
Dengue with warning signs^1^	–	4 (66.6%)	–	16 (44.4%)	0.400
Severe dengue^2^	–	0 (0%)	–	3 (8.3%)	1.000
Hemorrhagic manifestations^**3**^	–	4 (66.6%)	–	15 (41.6%)	0.384
Intravenous fluid resuscitation	–	1 (16.6%)	–	11 (30.5%)	0.655
Secondary Infection	–	6 (100%)	–	30 (83.3%)	1.000
PCR positive	–	4 (66.6%)	–	19 (52.7%)	0.673
DENV-1		0 (0.0%)		7 (36.8%)	0.567
DENV-4		4 (100%)		12 (63.3%)	0.180
IgM+	0 (0%)	6 (100%)	0 (0%)	31 (86.1%)	1.000
IgG+	2 (40%)	6 (100%)	11 (50%)	34 (94.4%)	1.00
NS1+	0 (0%)	2 (33.3%)	0 (0%)	6 (16.7%)	0.319

Data are expressed as median (interquartile range) or number (%).

* Patients whose platelets were used in the proteome analysis were also included in the validation cohort.

** *p* values between patients in proteome and validation cohorts.

^**1**^Abdominal pain or tenderness, persistent vomiting, clinical fluid accumulation, mucosal bleed or increased hematocrit concurrent with rapid decrease in platelet count; according to WHO criteria (2009).

^**2**^Severe plasma leakage, fluid accumulation with ascites (evidenced by ultrasonography), and/or severe bleeding (vaginal bleed and/or gastrointestinal bleed); according to WHO criteria (2009).

^**3**^Gingival bleed, vaginal bleed, gastrointestinal bleed, petechiae and/or exanthema.

ALT, alanine aminotransferase; AST, aspartate aminotransferase; TGO, glutamic-oxalacetic transaminase; TGP, glutamic-pyruvic transaminase.

Platelets from six patients with dengue (designated the dengue condition) and five healthy volunteers (designated the control condition) were digested with trypsin and fractionated with the isoelectric focalization of peptides (OFFGEL) system, generating 12 fractions. It was previously reported that OFFGEL fractionation, prior to MS analysis, enables identification of more peptides per protein particularly in low abundant molecules, and provides reliable results in both qualitative and quantitative levels [21,22]. Afterwards, a shotgun proteomic approach was applied (liquid chromatography tandem mass spectrometry–LC-MS/MS), where each OFFGEL fraction was analyzed on a high resolution mass spectrometer (Orbitrap XL) in technical triplicates. Quantification reproducibility was obtained according to normalized spectral abundance factors provided by PatternLab for Proteomics software. The MS raw files are available at http://max.ioc.fiocruz.br/supplementaryfiles/trugilho2016/ and readable by proteome analysis’ programs including the open source software PatternLab, Proteowizard or the Xcalibur from Thermo Fischer.

**Fig 1:**
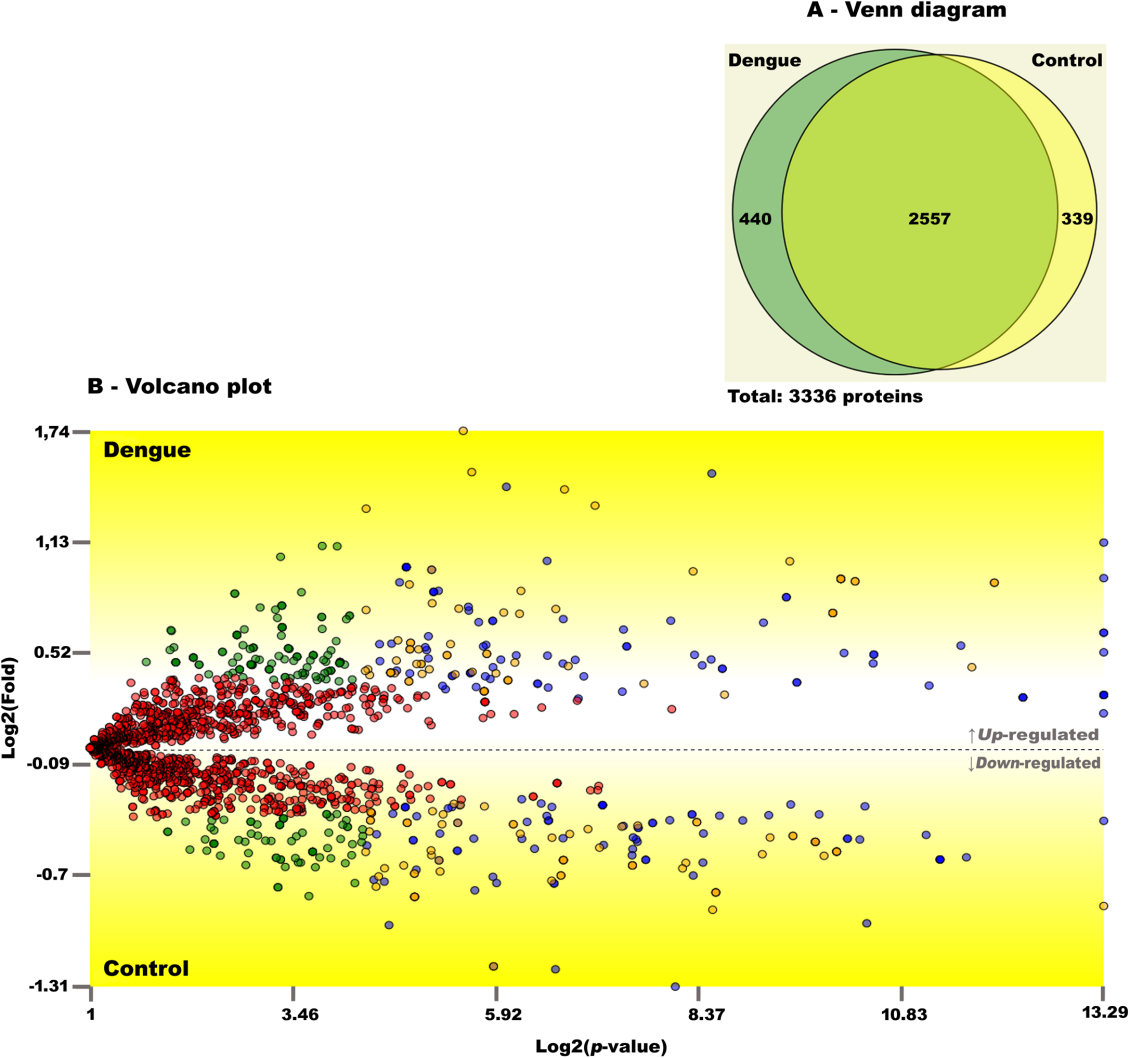
Distribution of mapped proteins in platelets from dengue patients and healthy volunteers. (**A**) Venn diagram of mapped protein entries present in platelets from healthy volunteers (Control) and patients with dengue. Both conditions shared 2,557 proteins; 339 and 440 proteins were identified only in control or dengue platelet samples, respectively. (**B**) Volcano plot of all shared protein entries and their abundance in dengue and control conditions. The graph represents TFold pairwise analysis of the two biological conditions. Each dot represents a protein mapped according to its log2 (fold change) on the ordinate axis and its -log2 (t-test p-value) on the abscissa axis. Red dots indicate proteins that satisfy neither the fold-change cutoff nor the FDR cutoff α (0.05). Green dots depict protein entries that satisfy the fold-change cutoff but not FDR α. Orange dots indicate proteins that satisfy both fold-change and FDR α, but present low fold changes. Blue dots represent protein entries that satisfy all statistical filters. The result shows 86 proteins significantly up-regulated (blue dots above the dotted line on Volcano plot) and 81 proteins down-regulated (blue dots below the dotted line on the graph) in platelets from patients with dengue.

Through these approaches, we were able to identify with high confidence (FDR < 1%) a total of 13,362 and 15,792 peptides in control and dengue samples, respectively, which infers up to 3,336 protein entries from Nextprot databank in both conditions (**Fig 1A** and **S1A** and **S1B Table**). There were no peptides from the DENV databank reliably detected in both conditions. Approximately 58% of proteins (1,956) were inferred from more than 4 peptides, and about 37% (1,236) had at least one proteotypic peptide observation (**S2B Table**). A simplified list of 1,777 proteins, according to the maximum parsimony criterion, is available in **S1D Table.** Dengue and control biological conditions shared 2,557 protein identifications; 440 and 339 proteins were uniquely detected in dengue and control platelet samples, respectively (**Fig 1A** and **S2A** and **S2B Table**).

Differentially abundant proteins were reported by Pattern Lab’s T Fold module (**Fig 1B**). One hundred and sixty-seven proteins showed statistically significant differences in their abundance between dengue and control platelets (**S2C Table**). As shown in **Fig 1B**, 86 proteins were significantly up-regulated while 81 proteins were significantly down-regulated in platelets from dengue patients compared to healthy volunteers.

Our differential abundance analysis considered proteins exclusively identified in each condition and at least in three replicates. This filtering process decreased the list of 440 and 339 proteins uniquely identified in dengue and control to 116 and 61, respectively (**S2A and S2B Table**). Although there are no p-values assigned to each protein, we argue this stringency criterion (i.e., being identified in more than one sample), plus the fact of only being identified in one biological condition, strongly suggests a differential abundance. As such, our final result shortlists the original 344 protein entries (167 shared proteins, 116 from dengue and 61 from control), down to 252 non-redundant entries (**S2D Table**).

### Differentially abundant proteins cluster in platelet activation and immune process pathways

**Fig 2:**
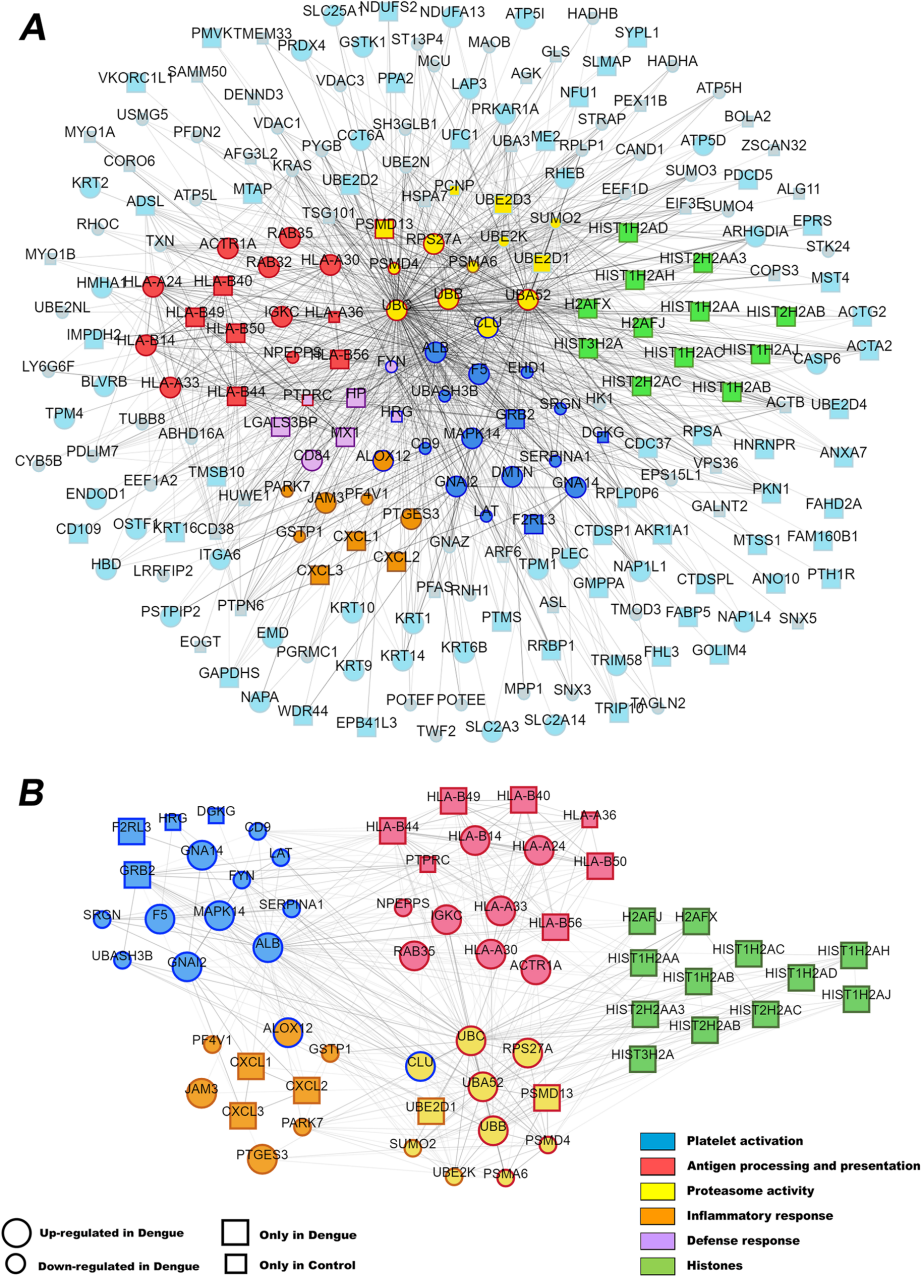
Interaction network of proteins differentially expressed between dengue and control platelets. (**A**) List of all 905 interactions between 252 differentially abundant proteins detected by the proteomic analysis. (**B**) A GO analysis validated the following biological processes: “platelet activation”, “antigen processing and presentation”, “proteasome activity”, “inflammatory response” and “histones”. Proteins labeled with two colors (fill and boundary colors) are involved in two biological processes. Larger circles represent proteins upregulated in dengue. Small circles indicate proteins downregulated in dengue. Squares represent proteins detected only dengue (large) or control (small) conditions.

We used the Search Tool for the Retrieval of Interacting Genes/Proteins (STRING) to generate a protein interaction map and categorize the differentially abundant proteins according to biological process classification in the Gene Ontology (GO) database. We reassembled the STRING network in the PINV software for improved visualization, facilitating data interpretation. The analysis of the 252 differentially expressed proteins revealed 905 possible interactions between 224 proteins. After generating the network graph (**Fig 2A**) we performed a GO analysis for biological processes. Although proteins in different biological processes overlapped, the most statistically enriched ones were assigned as “antigen processing and presentation” along with “platelet activation” (**S3 Table**). These enriched pathways, whose general GO identifiers are GO:0019882 (*p*-value = 2,73^−10^) and GO:0030168 (*p*-value = 8.36^−9^), respectively, were identified by the presence of at least fifteen exclusive differentially expressed proteins each. Interestingly, most of the proteins in the “antigen processing and presentation” pathway belong to HLA class I genes (colored in red, **Fig 2**). Other important biological processes highlighted were “protein polyubiquitination” (GO:0000209 with 14 related proteins and *p*-value = 5.88^−9^) and the closely related “proteasomal protein catabolic process” (GO:0010498 with 10 related proteins and *p*-value = 4.69^−5^). The proteins reported in these processes, named together as “proteasome activity”, directly interact with those in “platelet activation” (colors yellow and blue respectively, **Fig 2**). In addition, we found the GO terms “inflammatory response” and “defense response” with high significance and to be equally important. Finally, a cluster of histones was also distinguished (colored in green, **Fig 2A**). Importantly, most of the histones reported were histone H2A. A second protein interactions graph highlights these aforementioned relevant pathways (**Fig 2B**). The final list of differentially abundant proteins (**S2D Table**) together with the analysis of protein interaction maps obtained by STRING guided us to subsequent validation experiments.

### Platelet activation and secretion of granule stored chemokines in dengue

**Fig 3:**
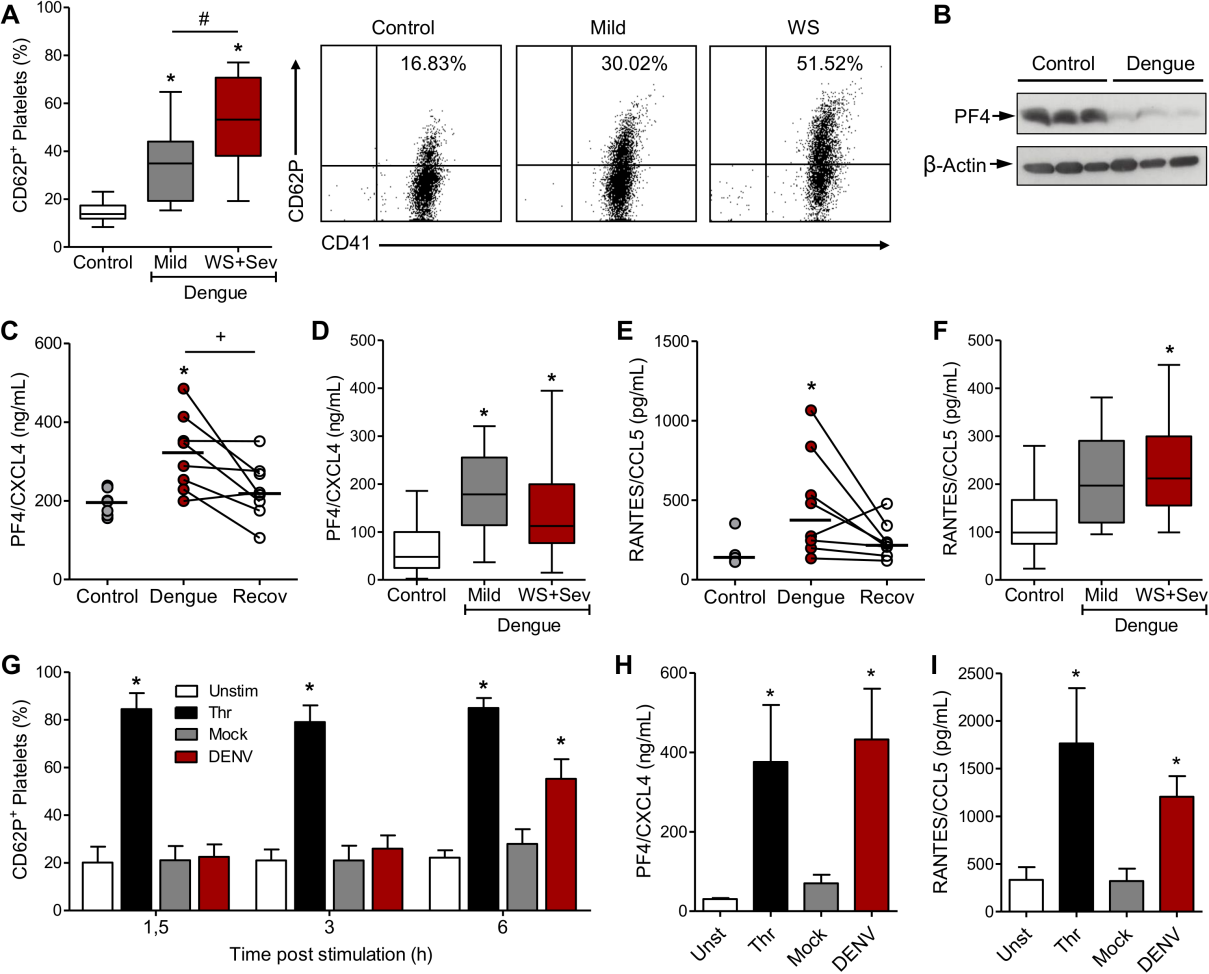
Dengue triggers platelet activation and chemokine secretion. (**A**) P-selectin (CD62P) surface expression by platelets isolated from healthy volunteers (Control) and patients with mild dengue, dengue with warning signs (WS) and severe dengue (Sev). (percentage of positive cells by flow cytometry). Boxes indicate the median and interquartile ranges and whiskers indicate minimal and maximal values in each group. Representative dot plots are shown. (**B**) Western blot analysis of platelet factor 4 (PF4/CXCL4) and β-actin in platelets isolated from three control subjects and three patients with dengue. (**C** and **E**) PF4/CXCL4 and RANTES/CCL5 concentration in supernatants of platelets (1 x 10^9^ per mL) from 8 control subjects and 8 dengue patients that were cultured for 4h *ex vivo*. Platelets obtained from the same patients in the recovery phase (Recov) were also evaluated. Each dot represents the level of chemokines secreted by platelets from one dengue patient or control. Horizontal lines represent median. (**D** and **F**) Concentration of PF4/CXCL4 and RANTES/CCL5 in plasma from control subjects and patients with dengue. Boxes indicate the median and interquartile ranges and whiskers indicate minimal and maximal values in each group. (**G-I**) Platelets isolated from healthy volunteers were kept unstimulated (Unst) or stimulated with thrombin (Thr), DENV or Mock for the indicated times. Panel **G** shows the percentage of CD62P-expressing platelets, and panels **H** to **I** show the concentration of PF4/CXCL4 and RANTES/CCL5 in the supernatant of platelets incubated in each condition for 6h. Bars represent mean ± standard error of the mean of 4 to 8 independent experiments performed with platelets from individual donors.* means p<0.05 compared to Control, Unst or Mock; # indicates p<0.05 between patients with mild and WS+Sev dengue syndromes; + represents p<0.05 between dengue patients in the acute and recovery phase (paired t test).

To investigate the relevance of the pathways identified in platelet proteome analysis to the pathophysiology of dengue, we validated the proteomic data in a cohort comprised by 36 dengue-infected patients with mild to severe dengue syndromes (**Table 1 and Table 2**). As shown in **Table 2**, dengue with warning signs and severe dengue patients had lower platelet counts, lower plasma albumin levels and higher frequencies of clinical signs of increased vascular permeability when compared to patients with mild dengue. We previously reported that platelets from dengue patients are activated [18]. Similarly, platelets from dengue patients included in this work were also activated as evidenced by P-selectin (CD62P) surface expression (**Fig 3A**). Importantly, platelet activation was higher in patients presenting dengue with warning signs and severe dengue syndromes compared to mild dengue patients (**Fig 3A**).

**Table 2:** Characteristics of dengue-infected patients classified as mild dengue or dengue with warning signs and severe dengue.

	Control (22)	Mild (17)	WS+Sev (19)	p*
Age, years	31 (29–34)	36 (29–49)	31 (22–41)	0.129
Gender, male	11 (50%)	8 (47%)	12 (63%)	0.503
Platelet count, x1,000 /mm^3^	–	121 (97.5–210.5)	98 (74–134)	**0.048**
Hematocrit, %	–	41.0 (39.5–44.2)	44.3 (39.6–46.7)	0.093
Albumin, g/dL	–	3.7 (3.6–3.9)	3.4 (3.1–3.7)	**0.034**
TGO/AST, IU/L	–	53.5 (28.7–92.0)	55.5 (34.5–131)	0.407
TGP/ALT, IU/L	–	91.5 (35.7–139.5)	64.0 (48.7–120)	0.985
Hemorrhagic manifestations^**1**^	–	5 (29.4%)	10 (52.6%)	0.192
Clinical signs of increased vascular permeability^2^	–	3 (17.6%)	15 (78,9%)	**<0.001**
Intravenous fluid resuscitation	–	1 (5.9%)	10 (52.6%)	**0.003**
Secondary Infection	–	15 (88.2%)	15 (78.9%)	0.662
PCR positive	–	9 (52.9%)	10 (52.6%)	1.000
DENV-1	–	3 (33.3%)	4 (40%)	1.000
DENV-4	–	6 (66.6%)	6 (60%)	1.000
IgM+	–	13 (76.5%)	18 (94.7%)	0.167
IgG+	–	17 (100%)	17 (89.5%)	0.487
NS1+	–	3 (17.6%)	3 (15.8%)	1.000

Data are expressed as median (interquartile range) or number (%).

^**1**^Gingival bleed, vaginal bleed, gastrointestinal bleed, petechiae and/or exanthema.

^2^Postural hypotension, oliguria, ascites, hypoalbuminemia (<3.6 g/dL) and/or >20%-increase in hematocrit.

*p values between mild dengue and dengue with warning signs plus severe dengue.

ALT, alanine aminotransferase; AST, aspartate aminotransferase; TGO, glutamicoxalacetic transaminase; TGP, glutamic-pyruvic transaminase.

The most representative protein entries in “inflammatory response” pathway were granule-stored chemokines (four out of eight protein entries, orange at **Fig 2**). We then analyzed the content of Platelet Factor 4 (PF4/CXCL4), a chemokine expressed exclusively by platelets and megakaryocytes that is stored in platelet alpha granules and secreted upon platelet activation [9]. PF4V1/CXCL4L1, a variant of PF4/CXCL4, was found to be down-regulated in platelets from dengue patients compared to control (**Fig 2B** and **S2D Table**). Likewise, western blot analysis of PF4/CXCL4 revealed lower PF4 quantities in platelets from patients with dengue compared to healthy volunteers (**Fig 3B**). Importantly, activated platelets from patients with dengue released higher levels of PF4/CXCL4 *ex vivo* when compared to control platelets (**Fig 3C**), despite reduced PF4 content (**Fig 3B**). These data suggest that platelets activated *in vivo* during dengue infection increasingly release granule-stored PF4/CXCL4, which may lead to its reduced intracellular content. Consistent with this, plasma levels of PF4/CXCL4 were increased in dengue-infected patients compared to healthy volunteers (**Fig 3D**). Similar results were observed for the granule-stored chemokine RANTES/CCL5 (**Fig 3E and 3F**).

Next, we investigated whether DENV infection directly induces platelet secretion of granule stored chemokines. Platelets from healthy volunteers were stimulated with DENV or mock culture medium *in vitro*, and thrombin-activated platelets were used as positive control (**Fig 3G**). DENV infection significantly increased the proportion of P-selectin-expressing platelets after six hours when compared to Mock-stimulated platelets (55.3 ± 21.7% for DENV versus 27.9 ± 16.2% for Mock, p = 0.002 paired t test) (**Fig 3G**). DENV infection also increased platelet secretion of the granule-stored chemokines PF4/CXCL4 and RANTES/CCL5 (**Fig 3H and 3I**). These data demonstrate DENV-triggered platelet translocation of granule stored factors and are in agreement with platelet degranulation *in vivo* during dengue infection, as suggested above.

### DENV increases HLA class I expression and surface display in platelets through proteasome dependent mechanisms

**Fig 4:**
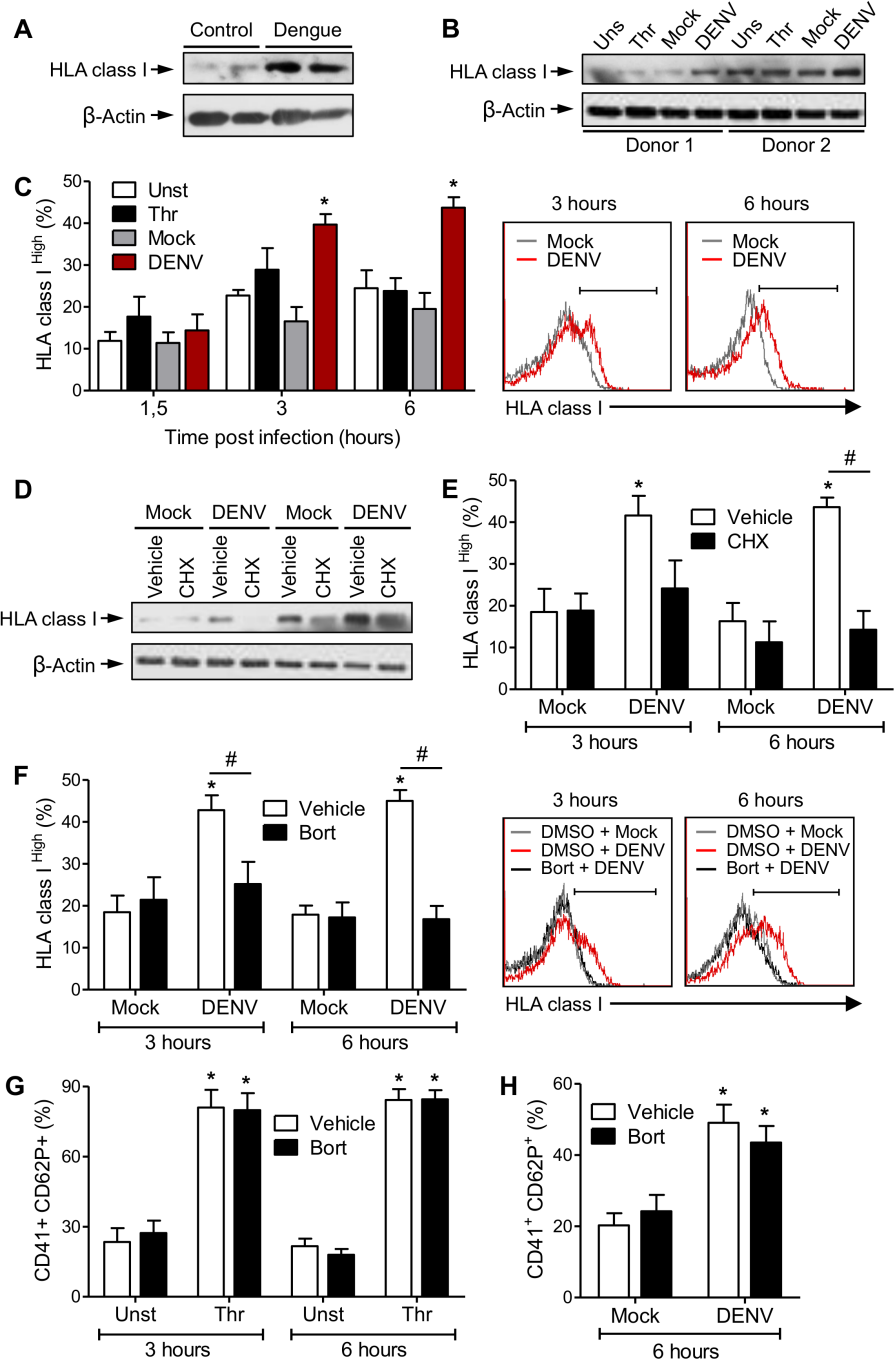
Increased HLA class I on DENV-infected platelets depends on protein translation and proteasome activity. **(A)** Western blot analysis for HLA class I and β-actin in freshly isolated platelets from two healthy control volunteers and two dengue-infected patients. (**B-H**) Platelets isolated from healthy volunteers were kept unstimulated (Unst) or stimulated with thrombin (Thr), DENV or Mock for the indicated times. Panel **B** shows the overall HLA class I expression in platelets from two independent donors at 6 hours post stimulation; and panel **C** shows the percent of platelets with high surface expression of HLA class I (HLA class I^High^) in each condition. (**D-H**) Platelets were exposed to DENV or Mock in the presence of DMSO (vehicle), bortezomib (1 μM) or cyclohexamide (10 μM). Panel **D** show the HLA class I expression at 6 hours post infection, panels **E**-**F** show the percent of HLA class I ^High^ expression and panels **G-H** depicts the P-selectin (CD62P) surface expression in platelets incubated in each condition. Bars represent mean ± standard error of the mean of 3 to 7 independent experiments from individual platelet donors. * indicates p<0.05 compared to unstimulated platelets or Mock; # means p<0.05 between platelets treated with Vehicle and Bortezomib or Cyclohexamide. Representative histograms are shown.

“Antigen processing and presentation” and “proteasome activity” were major activities identified based on differentially abundant proteins, with upregulation of HLA class I and proteasome subunits in platelets from dengue patients compared to healthy volunteers (**Fig 2B; S2D Table** and **S3 Table**). In agreement, HLA class I expression was increased in platelets from dengue patients compared to healthy volunteers when detected by western blot (**Fig 4A**). It has been shown by in-depth RNA sequencing that the mRNA for HLA class I subunits including b2-microglobulin and HLA are highly expressed in platelets [23]. Thus, we investigated if DENV infection *in vitro* increases HLA class I expression by platelets isolated from healthy uninfected donors. Platelets from healthy volunteers were stimulated with thrombin, mock or DENV, and HLA class I expression was evaluated by western blot. As shown in **Fig 4B**, DENV infection increased the expression of HLA class I in platelets. In contrast, platelet activation by thrombin did not affect HLA class I protein synthesis (**Fig 4B**). We next determined whether DENV infection enhances HLA class I trafficking and surface display. We observed a population of platelets that expressed significantly (p<0.05) higher levels of HLA class I on surface (HLA class I ^High^) at 3 and 6 hours post DENV infection compared to Mock (**Fig 4C**). Importantly, infection of platelets with DENV in the presence of the translational inhibitor cyclohexamide (10 μM), which inhibited HLA class I synthesis by platelets (**Fig 4D**), prevented platelets to increase HLA class I surface display in response to DENV (**Fig 4E**), suggesting that increased HLA class I synthesis is necessary before HLA class I trafficking to surface. To gain insights into the role played by proteasome protein processing in generating peptides for HLA class I presentation on platelets, platelets were treated with the proteasome inhibitor bortezomib (1 μM) for 30 min prior to DENV infection. Inhibition of proteasome activity prevented platelets to enhance HLA class I surface expression at 3 and 6 hours post DENV infection (16.8 ± 8.4% HLA class I^High^ for bortezomib versus 45.0 ± 6.9% for vehicle, p<0.005 paired t test, 6 hours post infection) (**Fig 4F**), suggesting that HLA class I loading by platelets depends on proteasome-generated peptides. Nevertheless, if the peptides presented derive from proteasome degradation of platelet or viral proteins remains unknown and should be further evaluated in the future.

To determine whether proteasome inhibition impaired HLA class I expression in a selective way, we evaluated the effects of bortezomib on thrombin- and DENV-triggered platelet activation. As shown in **Fig 4G and 4H**, treatment with bortezomib did not affect platelet P-selectin surface expression following thrombin-stimulation or DENV-infection, suggesting that inhibiting proteasome activity inhibits HLA class I surface display in a specific fashion.

### Platelets sequester histones released during dengue infection

**Fig 5:**
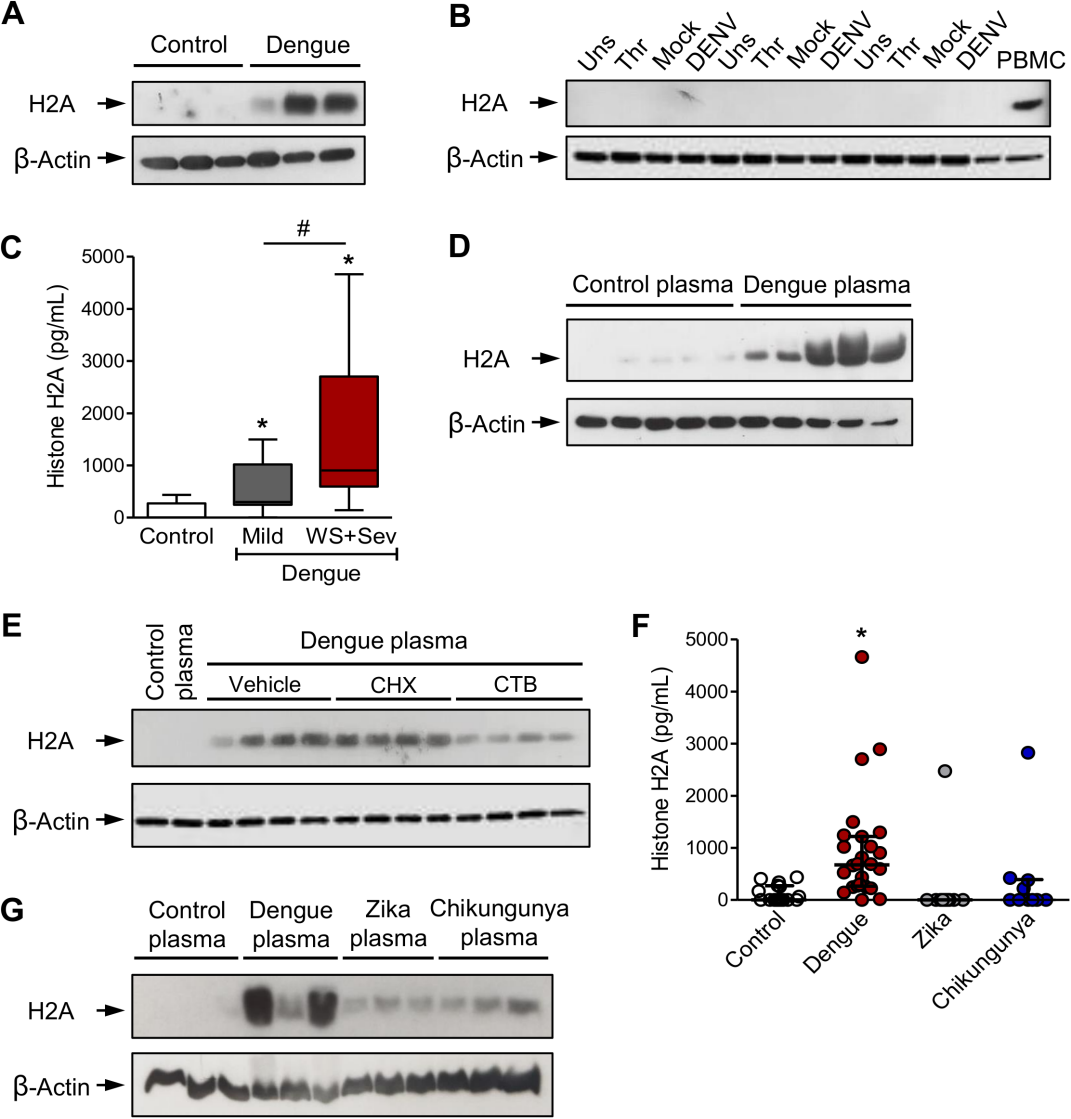
Platelets sequester circulating histone H2A in plasma from dengue-infected patients. (**A-B**) Western blot analysis for histone H2A and β-actin in (**A**) freshly isolated platelets from three healthy volunteers (Control) and three patients with dengue; and in (**B**) platelets from three healthy volunteers that were kept unstimulated (Unst) or stimulated with thrombin (Thr), DENV or Mock for 6h. Human peripheral blood mononuclear cells (PBMC) were used as positive control for histone H2A expression. (**C**) Histone H2A concentration in plasma from control subjects or patients with mild dengue or dengue with warning signs and severe dengue (WS+Sev). Boxes indicate the median and interquartile ranges and whiskers indicate minimal and maximal values in each group. (**D-E**) Platelets were isolated from a healthy volunteer and incubated with 20% plasma from five dengue-infected patients (dengue plasma) or five healthy volunteers (control plasma) for 4 hours in the presence or absence of cyclohexamide (CHX), cytochalasin B (CTB) or DMSO (vehicle). (**F**) Histone H2A concentration in plasma from control subjects or patients with dengue, zika or chikungunya fever. Each dot represents the level of histone H2A in plasma from one patient or control. Lines represent median and interquartile range. (**G**) Western blot analysis for histone H2A and β-actin in platelets incubated with 20% plasma from three control subjects or three patients with dengue, zika or chikungunya. * means p<0.05 compared to control, zika or chikungunya; # indicates p<0.05 between patients with mild and WS+Sev dengue syndromes. Western blots (**D, E** and **G**) are representative of three independent experiments from individual platelet donors.

In our proteomic analysis histones were detected with statistical confidence exclusively in platelets from dengue-infected patients (**Fig 2**and **S2D Table**). In agreement, histone H2A was detected by western blot in platelets from dengue patients but not in platelets from healthy volunteers (**Fig 5A**). In contrast, histones H2B and H3 were not detected in platelets from patients with dengue or in control platelets (**S1 Fig**). It was previously demonstrated by in-depth RNA sequencing that platelets from healthy volunteers have message RNA for all core histone subunits [23]. We then determined if DENV infection directly induces histone H2A synthesis by platelets. Platelets from healthy volunteers were stimulated with thrombin, mock or DENV *in vitro*, and histone H2A expression was evaluated. As shown in **Fig 5B**, DENV infection did not induce histone H2A expression by platelets, suggesting that histone H2A in platelets from dengue patients derives from its synthesis by infected megakaryocytes or from sequestration of free histones by platelets in the peripheral circulation.

We then measured the levels of histone H2A in plasma from patients with dengue and healthy volunteers and observed increased levels of circulating histone H2A during DENV infection (**Fig 5C**). In addition, higher levels of histone H2A were observed in plasma from dengue with warning signs and severe dengue patients compared to mild dengue (**Fig 5C**). To further explore the possibility that platelets are able to sequester histones circulating in plasma in dengue-infected patients, platelets from healthy volunteers were incubated (37°C at a 5% CO_2_ atmosphere) with 20% plasma from dengue-infected patients or from healthy volunteers. Platelet exposure to plasma from patients with dengue significantly increased their content of histone H2A when detected by western blot (**Fig 5D**). Inhibition of platelet protein translation by cyclohexamide (10μM) did not reduce platelet accumulation of histone H2A in response to dengue plasma whereas the cytoskeleton assembly inhibitor Cytochalasin B (1 μM), reduced the content of histone H2A protein (**Fig 5E**). These results demonstrate that histone H2A in platelets from dengue-infected patients may at least in part derive from the sequestration of circulating free histones by platelets.

Next, we evaluated whether platelet binding of circulating cell free histone H2A was a common feature among dengue and the related arbovirus diseases zika and chikungunya fever (**S4 Table**). Interestingly, we observed increased levels of circulating histone H2A in plasma from patients with dengue compared to zika or chikungunya patients (**Fig 5F**). Consistent with this observation, when platelets from healthy uninfected donors were incubated with plasma from patients with dengue, zika or chikungunya, higher content of histone H2A accumulated in platelets exposed to dengue plasma compared to zika or chikungunya (**Fig 5G**).

### Circulating histones in plasma from dengue patients activate platelets

**Fig 6:**
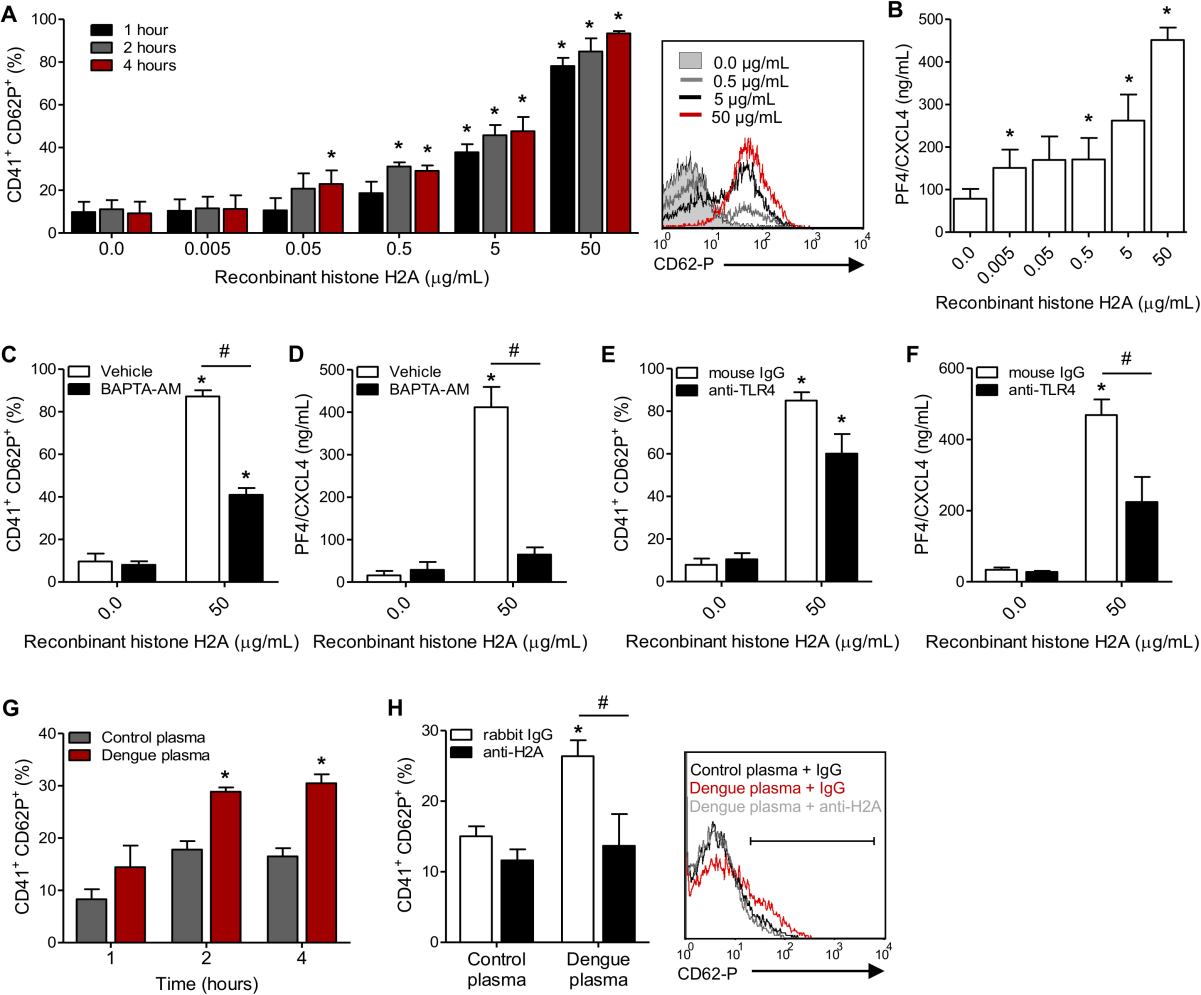
Circulating histone H2A in plasma from dengue-infected patients activates platelets. (**A-B**) Platelets isolated from healthy volunteers were stimulated with recombinant human histone H2A at the indicated concentrations. (**A**) Platelet surface P-selectin (CD62-P) was evaluated 1, 2 and 4 hours post stimulation by flow cytometry and (**B**) PF4/CXCL4 concentration was measured in supernatants 4 hours post stimulation. (**C-F**) Surface P-selectin and PF4/CXCL4 concentration in the supernatants of platelets stimulated with recombinant histone H2A for 2 hour in the presence of (**C-D**) the calcium chelator BAPTA-AM (20 μM) or vehicle (DMSO); or (**E-F**) blocking antibody against TLR4 (20 μg/mL) or isotype matched IgG. (**G-H**) P-selectin expression on platelets exposed to (**G**) plasma from six dengue-infected patients (dengue plasma) or four heterologous healthy volunteers (control plasma) for the indicated time-points; and (**H**) platelets exposed to dengue plasma or control plasma for 4 hours in the presence of anti-histone H2A (20 μg/mL) or isotype matched IgG. Bars represent mean ± standard error of the mean of 3 independent experiments (**A-F**) and of 4 to 6 independent plasma donors (**G-H**). * indicates p<0.05 compared to control plasma or unstimulated platelets; # represents p<0.05 between platelets treated with BAPTA-AM and vehicle or plasma samples treated with anti-histone H2A or isotype matched IgG. Representative histograms (**A** and **H**) illustrate surface P-selectin on platelets four hours after stimulation.

It has been previously shown that free histones bind to and activate platelets *in vitro* and *in vivo* [24,25,26,27]. However, the role played by circulating free histones in platelet activation during dengue infection remains unclear. Patients with dengue have increased levels of histone H2A in circulation (**Fig 5C**). We then investigated whether cell-free histone H2A is able to activate platelets *in vitro*. Treatment of platelets from healthy uninfected donors with recombinant human histone H2A significantly increased P-selectin translocation to the surface (**Fig 6A**) and secretion of PF4/CXCL4 into the supernatant (**Fig 6B**). Unfractionated histones have been shown to activate cellular responses through mechanisms involving toll like receptor (TLR) binding and calcium-mediated signaling [24,25,26,28,29]. Treatment of platelets with the calcium chelator BAPTA-AM (20 μM) significantly impaired P-selectin surface expression and PF4 secretion by histone H2A-activated platelets (**Fig 6C and 6D**). In addition, blocking of TLR4 significantly reduced platelet secretion of PF-4 and trended to reduce P-selectin translocation to platelet surface (**Fig 6E and 6F**), indicating that platelet activation by histone H2A partially depends on TLR4 binding. To investigate whether histone H2A in plasma from dengue patients is able to activate platelets, we incubated platelets from healthy volunteers with plasma from dengue-infected patients for 1, 2 and 4 hours, and observed increased platelet P-selectin surface expression in response to dengue plasma (**Fig 6G**). Next, we treated plasma from dengue infected patients and control plasma with rabbit IgG or anti-histone H2A (20 μg/mL) for 30 min prior to platelet stimulation. As shown in **Fig 6H**, blocking histone H2A prevented dengue plasma from inducing P-selectin translocation to the platelet surface.

## Discussion

Thrombocytopenia is a hallmark of dengue disease. Platelet count decline is temporally coincident with the critical phase of infection and correlates with the extension of hemodynamic instability and plasma leakage [6,7,30]. Although dengue pathophysiology is not completely elucidated, it has been shown that platelet activation plays a major role in inflammatory amplification and thrombocytopenia during dengue infection [18,19,20]. Considering this, our proteomic approach aimed to identify differentially abundant proteins in platelets from patients with dengue and matched healthy volunteers attempting to elucidate platelet activities during dengue illness. Our results reveal differentially expressed platelet proteins that point to immunoinflammatory platelet reprogramming in dengue-infected patients compared to healthy subjects. To gain insights into the platelet phenotype in dengue, we performed *in silico* analysis of protein interactions and gene ontologies and identified five main biological processes or components: “platelet activation”, “inflammatory response”, “antigen processing and presentation”, “proteasome activity” and “histones”. Finally, phenotypical and functional changes related to each of these processes were validated using platelet samples from a larger cohort of patients and by complementary mechanistic and functional assays.

Activation of circulating platelets in patients with dengue has been previously reported [18,20,31]. In our proteome analysis, proteins related to platelet activating signaling including PAR4 (F2RL3), G protein subunits (GNA12 and GNA14) and p38 MAPK (MAPK14) were increased in platelets from patients with dengue (blue dots in **Fig 2B and S2D Table**), potentially contributing to increased platelet activation during dengue infection. Consistent with these observations, dengue patients in the present study had increased P-selectin surface expression on platelets. In addition, P-selectin surface expression was increased in patients presenting dengue with warning signs and severe dengue syndromes compared to mild dengue (**Fig 3A**). P-selectin is a glycoprotein stored in platelet α-granules that is translocated to the surface and released in suspension during platelet activation [9]. It is the main adhesion molecule responsible for platelet interaction with monocytes [9,13,20,32], and circulating platelet-monocyte aggregates have been detected in dengue-infected patients [20,33]. Despite platelets from patients with dengue have increased P-selectin expression at baseline, it was recently shown that P-selectin trafficking to surface in response to thrombin receptor activating peptide stimulation *ex vivo* was lower in platelets from dengue patients compared to control [31], which is consistent with platelet exhaustion of α-granules proteins. Beyond P-selectin, platelet α-granules store numerous cytokines, chemokines and growth factors [9]. In agreement, we also observed exhaustion of the granule-stored chemokine PF4/CXCL4 in platelets from patients with dengue. Platelet exhaustion of PF4/CXCL4 content occurred in parallel with increased PF4/CXCL4 in plasma from dengue patients and PF4/CXCL4 secretion by platelets that were activated by DENV infection *in vitro* (**Fig 3B–3D**). A recent study reported that patients with severe dengue have lower levels of PF4/CXCL4 in plasma when compared to mild dengue patients [34]. This may result from lower platelet counts in patients with severe dengue, or from enhanced platelet exhaustion of PF4/CXCL4 content in severe dengue patients. More studies are still necessary to address the role played by platelet exhaustion in severity of dengue. Because of its roles in endothelial dissociation and angiogenesis [35], PF4 is potentially involved in vasculopathy of dengue syndromes.

Although increased P-selectin surface expression has been shown in platelets from dengue patients [18,31], the mechanisms underlying platelet activation in dengue are not completely understood. As shown here and in previous publications [18,36], platelets can be directly activated by DENV infection *in vitro* (**Fig 3G**). Platelet activation by DENV presents delayed kinetics of P-selectin expression compared to thrombin stimulation (**Fig 3G**) [18]. While “traditional” platelet activation by G-protein coupled receptors are rapid, it is now known that platelet activation in responses to infectious and immune stimuli, including LPS, can be delayed and sustained [9,37]. DENV activation of platelets requires infective DENV binding to DC-SIGN [18], a surface receptor involved in DENV binding and replication by platelets [17]. However, platelet activation in patients with dengue peaks at the critical phase of infection, when DENV particles are no longer circulating [18,31]. This indicates that other mechanisms are involved in platelet activation during nonviremic phases of dengue illness. Here we provide evidence that circulating histone H2A contributes to increased platelet activation in dengue (**Fig 6**). Histone H2A was detected exclusively in platelets from dengue infected patients, and higher levels of histone H2A were observed in plasma from dengue patients with warning signs and severe dengue. Histones can be released from necrotic cells and tissues or by neutrophils during the formation of neutrophil extracellular traps (NET), composed by released chromatin components (DNA and histones) and granule proteins [38,39,40]. Regarding this, DENV was recently shown to induce neutrophils to extend NETs *in vitro* [41]. Despite the fact that circulating histones have not been previously shown in dengue, a report showed that higher levels of circulating DNA associate with shock outcome in dengue-infected patients [42]. After exposing platelets to plasma from patients with dengue, we observed that histone H2A binds to and activates platelets (**Figs 5 and 6**). Similarly, when whole blood is exposed to histones *in vitro*, histones bind to platelets leading to platelet aggregation [26]. Accordingly, injection of histones into mice leads to histone accumulation in sites of thrombosis and to thrombocytopenia [24,26,27]. In experimental sepsis in mice and in patients with sepsis, a syndrome with many parallels with severe dengue, circulating histones are major mediators of vascular damage and disseminated intravascular coagulation (DIC) [27,29,43]. In addition to platelet activation, circulating free histones also activate endothelial cells amplifying the activation of inflammation and coagulation through endothelium expression of tissue factor and extrusion of Weibel-Paled bodies [24,28]. While the roles played by cell-free histones in vasculopathy, shock and organ dysfunction in dengue remain to be precisely determined, our observations strongly suggest that histone-mediated platelet activation may contribute to dengue pathogenesis.

In protein interaction analysis, the “antigen processing and presentation” pathway was closely associated with “proteasome activity” (red and yellow in **Fig 2,** respectively). The proteasome is a protein complex responsible for protein degradation in nucleated cells that has been previously reported to be present in platelets [44]. HLA class I proteins bind to and display on cell surface peptides from physiologic protein degradation by proteasome [45]. Through self-peptides presentation in HLA class I, nucleated cells and platelets survive cytotoxic T cell or NK cell immunosurveillance [45,46]. During viral or parasite infections, however, proteins from pathogens are also digested and presented by HLA class I, allowing cytotoxic T cell to eliminate infected cells [45]. A specific proteasome complex termed the immunoproteasome is constitutively expressed in immune cells and accelerate peptide generation for MHC class I antigen presentation, including during viral infections [45,47,48]. Recently, immunoproteasome subunits were reported to be present and functional in platelets [49]. Here, we showed that proteasome activity is required for increased HLA class I surface display in platelets following DENV infection (**Fig 4**). The ability of platelets to present exogenous antigens in HLA class I was recently demonstrated by Chapman and colleagues *in vitro* and in experimental model of cerebral malaria *in vivo* [14]. Platelets were able to activate T cell-mediated responses through HLA class I-mediated presentation of pathogen-derived antigens in that study [14]. Platelet-mediated T cell activation has demonstrated roles in immune activation and cytotoxic T lymphocyte-mediated platelet destruction [14,50,51,52,53] potentially contributing to cytokine storm and thrombocytopenia, both important pathogenic mechanisms of severe dengue [5,6,7,30,54]. Nonetheless, whether platelets process and present DENV-derived antigens in MHC class I and whether it impacts T cell activation and thrombocytopenia in dengue requires further investigation.

Several mechanisms can be involved in platelet proteome changes during natural DENV infection in humans. Even though platelets do not have nucleus, they have stored RNA molecules and diverse mechanisms for post transcriptional processing of RNA using specialized pathways to change their proteome, phenotype and function [9,23,55]. In addition, changes in platelet proteome observed in our study may result from alterations in megakaryocyte biology during dengue disease. It was previously demonstrated in nonhuman primates and in *ex vivo* infection of human marrow aspirates that megakaryocytes are the main target for DENV in marrow [56,57]. Even though we demonstrate that DENV infection increases HLA class I protein synthesis and surface display by platelets and that platelets sequester cell-free histones from dengue plasma, we recognize that alterations in platelet cargo during thrombopoiesis may contribute to these changes in the platelet proteome in dengue. In this regard, the CXC motif chemokines GRO1/CXCL1, MIP-2α/CXCL2 and GRO3/CXCL3 were detected exclusively in platelets from dengue patients by proteome (orange in **Fig 2B**), suggesting that dengue infection may change platelet granule’s protein content through a more inflammatory profile. While we took measures to deplete leukocytes from our platelet preparations, we were not able to completely eliminate leukocyte contamination. Nevertheless, our proteome analysis revealed that the leukocyte marker CD45 (PTPRC) was detected only in control samples (purple in **Fig 2A, S2 Table**), excluding leukocyte contamination as a determinant for increased HLA class I, histones or chemokines expression in platelets from dengue patients.

**Fig 7:**
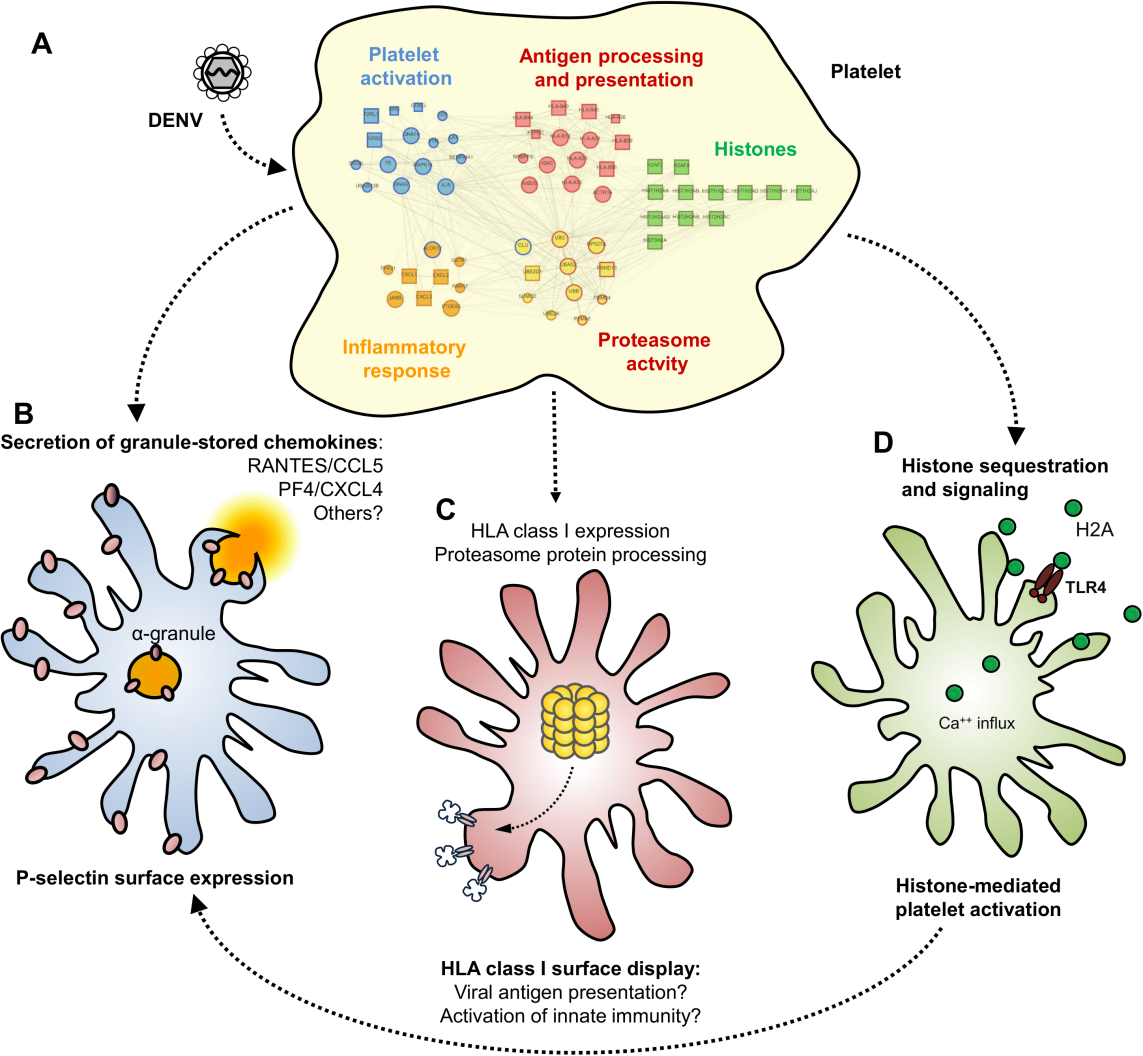
Schematic representation of proteome changes in platelets from dengue-infected patients. (**A**) Platelets from dengue-infected patients exhibit changes in their proteome, phenotype and function. Alterations in the platelet proteome in dengue appear to influence five main biological processes based on our analysis: (i) platelet activation; (ii) antigen processing and presentation; (iii) processes influenced by proteasome dependent protein catabolism; (iv) inflammatory response and; (v) histone expression and signaling. (**B**) DENV infection activates platelets triggering granule release of stored chemokines (i.e. PF4/CXCL4 and RANTES/CCL5) and P-selectin surface expression. (**C**) DENV also increases HLA class I protein expression and its surface presentation through mechanisms requiring proteasome protein processing. The nature of peptides processed by proteasome activity and presented in HLA class I remains unknown. (**D**) Platelets sequester circulating cell-free histone H2A from plasma, which contributes to platelet activation during dengue infection.

Emerging evidence identifies platelets as dynamic cells that represent a link between inflammation and pro-thrombotic responses in many vascular and inflammatory processes [9,10,11,58]. Consistent with this notion, our findings provide novel biological evidence that platelets undergo dynamic changes in dengue resulting in phenotypic changes implicated in immune and inflammatory processes that are of recognized relevance to dengue pathophysiology (Summarized in **Fig 7**). Platelets may be activated during dengue illness by parallel or sequential mechanisms, which may include direct DENV infection of platelets as well as indirect activation resulting from platelet signaling by sequestered circulating histones. This infection-driven reprograming of platelets in dengue alters the regulation of HLA class I expression on platelets and the secretion of cytokines and chemokines. Thus, platelets can affect the immune and inflammatory milieu of dengue illness, with potential consequences to disease progression and severity. These functional changes demonstrated in platelets from patients and *in vitro* experiments in this report, and others that may be discovered from our analysis of the platelet proteome in dengue patients, will contribute to a better understanding of platelet activities in dengue pathogenesis.

## Materials and methods

### Human subjects

Peripheral vein blood samples were obtained from thirty-six serologically/molecularly confirmed DENV-infected patients examined at the Instituto Nacional de Infectologia Evandro Chagas (INI)–Fundação Oswaldo Cruz, Rio de Janeiro, Brazil, during the dengue outbreaks of 2011–2013. Clinical and laboratorial characteristics of dengue-infected patients are presented in **Tables 1**and **2**. Samples were collected on an average of 4.4±1.8 days after onset of illness and first symptom presentation. Peripheral vein blood was also collected from ten patients with zika virus infection and ten patients with chikungunya fever examined at the Quinta D’or hospital in 2016 (**S4 Table**); and from twenty-two age-matched healthy subjects.

Dengue-infected patients were classified according to the World Health Organization (WHO) dengue case definition guideline [3] as having mild dengue (47.2%), dengue with warning signs (44.4%) or severe dengue (8.3%). Diagnosis of dengue patients (which were included before zika and chikungunya virus introduction in Brazil) was confirmed as clinical symptoms and signs consistent with dengue with positive plasma detection of DENV genome, IgM antibodies against DENV E protein and/or DENV NS1 antigen. All zika and chikungunya infected patients had the diagnostic confirmed by detection of zika virus (ZIKV) or chikungunya virus (CHIKV) genome, respectively. For viral RNA detection and typing, viral RNA was extracted (QIAamp Viral RNA mini kit, Quiagen) from plasma samples and processed as previously described [59,60]. Levels of IgM and IgG specific for DENV E protein were measured using standard capture ELISA Kit according to the manufacturer’s instructions (PanBio). DENV NS1 protein was detected in patient plasma using the NS1 detection Kit (BioRad). Primary and secondary infections were distinguished using IgM/IgG antibody ratio as previously described [61,62,63]. Five of six included patients were diagnosed with a secondary dengue infection (**Tables 1 and 2**).

### Ethics statement

The study protocol was approved by the Institutional Review Board (INI # 016/2010 and IOC/FIOCRUZ # 42999214.1.1001.5248) and the experiments were performed in compliance with this protocol. Written informed consent was obtained from all volunteers prior to any study-related procedure in accordance with the Declaration of Helsinki.

### Platelet isolation

Peripheral blood samples were drawn into acid-citrate-dextrose (ACD) and centrifuged at 200 x g for 20 minutes to obtain platelet-rich plasma (PRP). Platelets were isolated from PRP and CD45+ leukocytes were depleted from platelet preparations as previously described [19,20]. Briefly, PRP was centrifuged at 500 xg for 20 min in the presence of 100 nM Prostaglandin E_1_ (PGE_1_) (Cayman Chemicals). The supernatant was discarded, and the platelet pellet was resuspended in 2.5 mL of phosphate-buffered saline containing 2 mM EDTA, 0.5% human serum albumin and 100 nM PGE_1_ and incubated with anti-CD45 tetrameric antibody complexes (1:25) for 10 minutes and with dextran-coated magnetic beads (1:50) for additional 15 minutes before purification in a magnet (Human CD45 depletion kit; StemCell, Easy Sep Technology). Recovered platelets were resuspended in 25 mL of PSG (PIPES-saline-glucose: 5 mM C_8_H_18_N_2_O_6_S_2_, 145 mM NaCl, 4 mM KCl, 50 mM Na_2_HPO_4_, 1 mM MgCl_2_-6H2O, and 5.5 mM glucose) containing 100 nM of PGE_1_. The platelet suspension was centrifuged at 500 xg for 20 minutes. The supernatant was discarded and the pellet resuspended in medium 199 (Lonza). The purity of the platelet preparations (>99% CD41+) was confirmed by flow cytometry.

### Sample preparation

Isolated platelets from each dengue patient (n = 6) or control subject (n = 5) were individually assessed by proteome analysis as follows. Platelets (1 x 10^8^) were centrifuged at 700 x *g* for 10 min and resuspended in 50 mM NH_4_HCO_3_ containing 0.2% of RapiGest SF (Waters) for cell lysis. The protein concentration was determined in each individual sample using the bicinchoninic acid assay (BCA) according to the manufacturer's instructions (Sigma-Aldrich). Each sample (100 μg of protein), was reduced with dithiothreitol to a final concentration of 5 mM for 3 h at 37°C. After reaching room temperature, the samples were alkylated with iodoacetamide to a final concentration of 15 mM for 15 min while protected from light. Trypsin (Promega, USA) was added in a 1:50 (p/p) enzyme/substrate ratio. The digestion was performed for approximately 24 h at 37^°^C, and reaction was stopped with 1% formic acid. Aliquots of 50 μg of the initial digests were quantitated by Nanodrop spectrophotometry at 280 nm (Thermo Fisher Scientific), desalted with POROS R2 resin (Applied Biosystems), packaged in micropipette tips (Millipore, Bedford, USA) and equilibrated in TFA 1%. After washing with TFA 0.1%, the peptides were eluted in TFA 0.1% containing acetonitrile 70% and completely dried in vacuum centrifuge.

### Isoelectric focalization of peptides (OFFGEL)

Peptides were solubilized with peptide OFFGEL solution and separated using an Agilent 3100 OFFGEL Fractionator with OFFGEL High Res Kit, pH 3–10 immobilized pH gradient (IPG) DryStrips according to manufacturer’s instructions (Agilent Technologies, Germany). Twelve well fractionations were focused for 20 kVh with a maximum current of 50 mA and power of 200 mW. Each fraction was separately desalted, as described in the previous section, and suspended in 40 μL of 1% formic acid.

### Liquid chromatography-tandem mass spectrometry (LC-MS/MS) analysis

The desalted peptides from each OFFGEL fraction were loaded onto a 10 cm reversed phase (RP) column and separated on-line to the mass spectrometer by using Easy nLC II (Thermo Scientific). Four microliters were initially applied to a 2-cm long (100 μm internal diameter) trap column packed with 5 μm, 200 A Magic C18 AQ matrix (Michrom Bioresources, USA) followed by separation on a 10-cm long (75 μm internal diameter) separation column. Samples were loaded onto the trap column at 2000 μL/min while chromatographic separation occurred at 200 nL/min. Mobile phase A consisted of 0.1% (v/v) formic acid in water while mobile phase B consisted of 0.1% (v/v) formic acid in acetonitrile. Peptides were eluted with a gradient of 2 to 40% of B over 32 min followed by up to 80% B in 4 min, maintaining at this concentration for 2 min more, before column reequilibration. The HPLC system was coupled to the LTQ-Orbitrap XL via a nanoscale LC interface (Thermo Scientific). Source voltage was set to 1.9 kV, and the temperature of the heated capillary was set to 200^°^C and tube lens voltage to 100 V. MS1 spectra were acquired on the Orbitrap analyzer (300 to 1,700 m/z) at a 60,000 resolution (FWHM at m/z 400). FTMS full AGC target was set to 500,000 and ion trap MSn AGC target was set to 30,000. For each survey scan, the 10 most intense ions were submitted to CID fragmentation (minimum signal required of 10,000; isolation width of 2.5 m/z; normalized collision energy of 35.0; activation Q of 0.25 and activation time of 30 s) followed by MS2 acquisition on the linear trap analyzer. Dynamic exclusion option was enabled and set with the following values for each parameter: repeat count = 1; repeat duration = 30 s; exclusion list size = 500; exclusion duration = 45 s and exclusion mass width = 10 ppm. Data were acquired in technical triplicates using the Xcalibur software (version 2.0.7).

### Computational analysis

The raw data files were processed and quantified using PatternLab for Proteomics software (http://www.patternlabforproteomics.org/) [64]. Peptide sequence matching (PSM) was performed using the Comet algorithm [65] against the protein-centric human database NeXtProt [66] (manually annotated and recommended by HUPO—Human Proteome Organization) plus a FASTA file containing DENV sequences retrieved from the NCBI database (GeneBank taxon number 14,164). A target-reverse strategy was employed. The search considered tryptic and semi-tryptic peptide candidates. Cysteine carbamidomethylation and oxidation of methionine were considered as fixed and variable modifications, respectively. The Comet search engine considered a precursor mass tolerance of 40 ppm and bins of 1.0005 for the MS/MS. The validity of the peptide spectrum matches were assessed using PatternLab’s Search Engine Processor (SEPro) module [67]. Identifications were grouped by charge state (+2 and > +3) and then by tryptic status (semi-tryptic), resulting in four distinct subgroups. For each result, the XCorr, DeltaCN and ZScore values were used to generate a Bayesian discriminator. SEPro then automatically established a cutoff score to accept a false-discovery rate (FDR) of 1% based on the number of decoys, independently performed on each data subset, resulting in a false-positive rate that was independent of tryptic status or charge state. Additionally, a minimum sequence length of 6 amino acid residues was required. Similar proteins, which represent an identical sequence and consist of a fragment of another sequence, were eliminated. Then, only PSMs with less than 5 ppm were considered to compose a final list of mapped proteins supported by at least three independent characteristics (e.g., identification of a peptide in charge states, modified and non-modified version of the same peptide, or different peptides). All identification results are reported with less than 1% FDR both in peptide and protein levels. Spectral counting were used as a surrogate for semi-quantitation according to the normalized spectral abundance factor (NSAF) [68]. Differentially abundant proteins were pinpointed using PatternLab’s TFold module with a Benjamini–Hochberg q-value of 0.05 [69]. The approximately area-proportional Venn diagram module displayed all mapped proteins in each condition.

Protein interaction networks for differentially abundant proteins were developed using the STRING database (http://string-db.org/) [70]. Enrichment analysis for biological processes annotation was performed using the Gene Ontology (GO) databank available as a tool inside STRING. The generated networks were edited according to the GO terms classification and submitted to the Protein Interaction Network Visualizer—PINV (http://biosual.cbio.uct.ac.za/pinv.html) [71].

### In vitro platelet stimulation

DENV serotype 2 strain 16881 was propagated in C6/36 *Aedes albopictus* mosquito cells and titrated by plaque assay on BHK cells [72]. The quantity of infectious particles was expressed as plaque forming units (PFU)/mL. Platelets from healthy uninfected donors were incubated (37°C in a 5% CO_2_ atmosphere) with DENV-2 at a multiplicity of infection of 1 PFU/ platelet, with thrombin (Sigma, T1063) (0,5 U/mL) or with recombinant human histone H2A (BioLabs, M2502S) for the indicated times. Supernatants from uninfected C6/36 cell cultures (mock) were produced using the same conditions and used as a control for platelet stimulation by DENV. To characterize the mechanisms involved in platelet surface expression of HLA class I, we pre-incubated platelets with the proteasome inhibitor bortezomib (LC Laboratories, MA) (1 μM) or the translational inhibitor cyclohexamide (10μM) for 30 min prior to DENV infection.

Platelets from healthy volunteers were incubated (37°C in a 5% CO_2_ atmosphere) with plasma from dengue-infected patients or heterologous healthy volunteers for the indicated times. To characterize the role played by circulating histones in platelet activation, plasma samples were treated with anti-histone H2A (Santa Cruz sc-10807) (20 μg/mL) for 30 min prior platelet stimulation. To characterize the mechanisms involved in platelet activation by cell free histone H2A, platelets were pretreated with the calcium chelator BAPTA-AM (Sigma) (20 μM) or anti-TLR4 neutralizing antibodies (eBioscience 169917–82) (20 μg/mL) for 30 min prior stimulation with histone H2A.

### Flow cytometry analysis

Platelets (1–5 x 10^6^) were incubated with FITC-conjugated anti-CD41 (BD Phamingen, CA) (1:20), PE-conjugated anti-CD62-P (BD Pharmingen, CA) (1:20) and APC-conjugated anti-HLA-A, B, C (Biolegend, CA) (1:50) for 30 min at 37°C. Isotype-matched antibodies were used to control nonspecific binding of antibodies. Platelets were distinguished by specific binding of anti-CD41 and characteristic forward and side scattering. A minimum of 10,000 gated events were acquired using a FACScalibur flow cytometer (BD Bioscience, CA).

### Western blotting

*In vitro* stimulated platelets or freshly isolated platelets from dengue patients and healthy volunteers were lysed (0.15 M NaCl, 10mM Tris pH 8.0, 0.1 mM EDTA, 10% Glicerol and 0.5% triton X-100) in the presence of protease inhibitors (Roche, Indianapolis, IN). Platelet proteins (20 μg) were separated by 15% sodium dodecyl sulfate-polyacrylamide gel electrophoresis (SDS–PAGE) and transferred into nitrocellulose membranes. Membranes were blocked in Tris-buffered saline (TBS) supplemented with 0.1% Tween 20 (TBS-T) plus 5% milk for 1 h before incubation overnight with primary mouse anti-human PF4 (R&D Systems) (1:500) or rabbit-anti-human Histone H2A (Cell Signaling) (1:1000) or biotinylated-mouse-anti-human HLA class I (eBioscience) (1:1000), or for 1 h with mouse anti-human β-actin (Sigma Aldrich) (1:20,000) antibodies. After washing five times in TBS-T, membranes were revealed using fluorescent dye-conjugated or peroxidase-conjugated secondary antibodies (Vector) (1:10,000) or streptavidin (R&D) (1:200).

### Chemokines and histone H2A measurement

Washed platelets (10^9^ per mL) isolated from eight healthy volunteers or eight dengue-infected patients were incubated at 37°C in a 5% CO_2_ atmosphere. After 4 hours of incubation, the platelets were pelleted, the supernatants were harvested and the secreted levels of PF-4/CXCL4 and RANTES/CCL5 were measured using standard ELISA protocol according to manufacturer’s instructions (R&D systems). PF4/CXCL4 and RANTES/CCL5 were also measured in supernatants of platelets obtained from the same patients at the recovery phase (average 17.5±6.5 days after the onset of illness).

Plasma samples were collected from ACD-anticoagulated blood from patients and healthy volunteers and frozen in liquid nitrogen until use. PF4/CXCL4 plasma levels were quantified in 1:200 diluted samples. Circulating histone H2A was measured using a standard ELISA protocol according to manufacturer’s instructions (LSBio, LS-F238).

### Statistical analysis

Complementary statistics was performed using GraphPad Prism, version 5.0 (GraphPad, San Diego, CA). The numerical demographic and clinical variables are expressed as median and interquartile range (25–75 percentile) or as number and percentage (%). All numerical variables were tested for normal distribution using the Kolmogorov-Smirnov test. For comparisons among three groups we used Oneway ANOVA to determine differences and Bonferroni’s multiple comparison test to locate the differences among groups. For comparisons between two groups we compared the continuous variables using the t test for parametric distribution or the Mann–Whitney U test for nonparametric distribution. The paired two-tailed t-test was used to compare *in vitro* stimulated platelets with unstimulated platelets from the same donor. Qualitative variables were compared by the two tailed Fisher test using Epi-Info software version 7.0 (CDC).

## Supporting information

S1 Fig Western blot analysis for histone H2A, histone H2B, histone H3 and β-actin in freshly isolated platelets from three healthy volunteers (Control) and three patients with dengue. (PDF)

S1 Table (**A**) Absolute numbers of all peptides and proteins identified in platelet samples. (**B**) List of all peptides confidently identified in the platelet proteome. (**C**) List of all proteins confidently identified in the platelet proteome. (**D**) All proteins confidently identified in platelet samples with maximum parsimony criterion.
(XLSX)

S2 Table (**A**) List of the proteins detected only in Control biological condition. (**B**) List of the proteins detected only in Dengue biological condition. (**C**) List of differentially expressed proteins detected in both biological conditions (Dengue and Control) with statistical significance (FDR < 0.05). These proteins represents the blue dots in volcano plot (Fig 1B). (**D**) List of all differentially expressed proteins, include the proteins detected in only one biological condition (at least 3 technical replicates). The function description was extracted from the Platelet Web (http://plateletweb.bioapps.biozentrum.uni-wuerzburg.de/plateletweb.php) or NextProt (https://www.nextprot.org).
(XLSX)

S3 Table (**A**): List of all gene ontology terms generated from the list of differentially abundance proteins. Extracted from STRING software—GO Biological Processes tool (http://string-db.org/).
(XLSX)

S4 Table Characteristics of patients with dengue, zika or chikungunya infection. (DOCX)
