# Supplementary figures and images for: Platelet proteome reveals novel pathways of platelet activation and platelet-mediated immunoregulation in dengue
Source: PLoS Pathog. 2017 May 19;13(5):e1006385. doi: 10.1371/journal.ppat.1006385 (PMC5453622; doi:10.1371/journal.ppat.1006385)

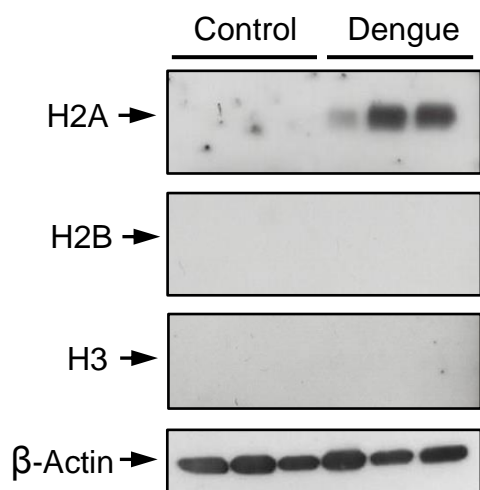

Supplement: S1 Fig — (PDF) [file ppat.1006385.s001.pdf]
