# Supplementary material for: Platelet proteome reveals novel pathways of platelet activation and platelet-mediated immunoregulation in dengue
Source: PLoS Pathog. 2017 May 19;13(5):e1006385. doi: 10.1371/journal.ppat.1006385 (PMC5453622; doi:10.1371/journal.ppat.1006385)
Supplement: S4 Table — (DOCX) [file ppat.1006385.s005.docx]

**Supplemental Table 4:** Characteristics of patients with dengue, zika or chikungunya infection.

|  | **Control (22)** | **Dengue (36)** | **Zika (10)** | **Chikungunya (10)** |
| --- | --- | --- | --- | --- |
| Age, years | 31 (29-34) | 33 (28-42) | 33 (31-36) | 35 (34-47) |
| Gender, male | 11 (50%) | 20 (55.5%) | 5 (50%) | 5 (50%) |
| Day of blood draw^1^ | – | 4 (3-5) | 2 (2-4.5) | 3 (2-4.3) |
| Platelet count, x1,000 /mm^3^ | – | 109 (80-161) | 210 (181-230)* | 149(104-210) |
| Hematocrit, % | – | 41.8 (39.7-45) | 39.7 (36-47) | 41.4 (39-44) |
| Petechiae/ exanthema | – | 11 (30.5%) | 6 (60%) | 3 (30%) |
| Clinical signs of increased vascular permeability^2^ | – | 18 (50%) | 0 (0.0%)* | 0 (0.0%)* |

Data are expressed as median (interquartile range) or number (%).

^1^Days after onset of illness and first symptom presentation.

^2^Postural hypotension, oliguria, ascites, hypoalbuminemia (<3.6 g/dL) and/or >20%-increase in hematocrit.

*p<0.05 when compared to dengue.
